# The role of common variants of the cholesteryl ester transfer protein gene in left main coronary artery disease

**DOI:** 10.1186/1476-511X-10-156

**Published:** 2011-09-07

**Authors:** Genovefa Kolovou, Ioannis Vasiliadis, Vana Kolovou, Agathi Karakosta, Sophie Mavrogeni, Evaggelia Papadopoulou, Spiridon Papamentzelopoulos, Vasiliki Giannakopoulou, Apostolia Marvaki, Dimitrios Degiannis, Helen Bilianou

**Affiliations:** 1 1st Cardiology Department, Onassis Cardiac Surgery Center 356, Sygrou Ave., 176 74 Athens, Greece; 2 Molecular Immunology Laboratory, Onassis Cardiac Surgery Center 356, Sygrou Ave., 176 74 Athens, Greece; 3 Thriasio General Hospital, Genimata Ave., 19600 Magoula, Attiki, Greece; 4 Tzaneio Hospital,18536 Piraeus, Greece

**Keywords:** Left main coronary artery disease, atherosclerosis, TaqIB polymorphism, I405V polymorphism.

## Abstract

**Background:**

The cholesteryl ester transfer protein (CETP) has a central role in the lipid metabolism and therefore may alter the susceptibility to atherosclerosis.

**Methods:**

The DNA of 471 subjects [133 subjects with angiographically documented left main coronary artery disease (LMCAD), 241 subjects with more peripheral coronary artery disease (MPCAD) and 97 subjects self reported healthy (Controls)] was analyzed for the frequency of TaqIB and I405V polymorphisms in the gene coding CETP.

**Results:**

There is no significant difference in CETP allele frequency or genotype distribution among LMCAD and MPCAD patients although there is statistical difference between LMCAD and Controls (p = 0.001). Specifically, patients with LMCAD and B1B1 genotype of TaqIB polymorphism were more frequent present compared to Controls (33.8% vs 22.9%, respectively). The frequency of B2B2 genotype was 3 times lower in the LMCAD group compared to Controls (10.5% vs 30.2%, respectively). In the LMCAD group the frequency of B1 allele compared to Controls was higher (62% vs 46%, respectively, p = 0.001). The relationship between TaqIB gene polymorphism and the LMCAD was independent of lipid profile, with the exception of apolipoprotein A.

**Conclusions:**

These findings indicate that the TaqIB polymorphism may have potential importance in screening individuals at high risk for developing CAD. However, this polymorphism cannot distinguish between LMCAD and MPCAD. Further prospective investigations in larger populations are required to confirm these findings.

## Introduction

The evolution of coronary artery disease (CAD) is influenced by various genetic and environmental factors. The genetic contribution is documented by a positive family history for myocardial infarction and is considered to be a strong cardiovascular risk factor [1,2]. This has been supported even after adjustment for classical risk factors such as diabetes mellitus, dyslipidemia, hypertension and others [3-5]. Furthermore, the level of high density lipoprotein cholesterol (HDL-C) in plasma is a major determinant of susceptibility to coronary atherosclerosis [6-8]. Genetic studies have recognized the impact of genetic mutations on plasma HDL-C levels. Such one example is Tangier disease [9,10], where very low plasma HDL-C levels may lead to premature coronary atherosclerosis in spite of very low low density lipoprotein cholesterol (LDL-C) levels. It seems that the heritability of plasma HDL-C level is likely to be higher than 50% [11-13]. The estimates of heritability of plasma HDL concentration in the Strong Heart Family Study [13] and HERITAGE family study [12] were 50% and 52%, respectively. However, the genome-wide association studies (GWAS) accounted for only 5-8% of the variation in the plasma HDL-C levels [14,15]. Despite extensive molecular genetics investigations, non specific reproducibly genetic variants associated with CAD were found. This can be explained by the multifaceted phenotype of CAD (number of involved vessels, location of lesions, severity of diameter narrowing, length and morphology of lesions), suggesting different mechanisms. The CAD involving left main (LM) artery is a particular severe phenotype of CAD. Thus, the LM stenosis localized patients at the higher risk of cardiovascular events because of the extent of jeopardized myocardium and, therefore, has been considered as the most prognostically important coronary lesion. Fischer et al [16] reported a stronger genetic component in this phenotype compared to CAD involving more peripheral (MP) coronary arteries. Furthermore, this phenotype of CAD should probably provide more power in detecting genetic association. Since the discovery of cholesteryl ester transfer protein (CETP) and its identification as a modulator of HDL-C levels (mediates the exchange of lipids between lipoproteins), there has been much speculation about its role in CAD [17]. Studies with animal models have been limited, because many species do not express a functional CETP protein and are not reliable to provide strong evidence for CETP's role in disease. Thus the studies evaluating the influence of CETP gene polymorphisms in humans are essential. The CETP gene is located on chromosome 16q21. Widely studied CETP variants is a silent base change called the Thermobius aquaticus IB (TaqIB) affecting the 277th nucleotide in the first intron of the CETP gene [18]. In normolipidemic subjects, the absence of the TaqIB restriction site (B2 allele) is associated with decreased CETP activity and, in turn, increased HDL-C levels [19] resembling a mild form of CETP deficiency. In line with our previous work, using the LMCAD phenotype, we investigated whether the common polymorphisms (TaqIB and I405V) of CETP gene are able to identify LMCAD from MPCAD or from Controls.

## Methods

The DNA of 471 subjects of Greek origin, not related was analyzed for the presence of TaqIB and I405V polymorphisms in the gene coding for CETP.

Subjects were consecutively recruited among those admitted to hospital for coronary angiography. The Onassis Cardiac Surgery Center is a major referral hospital for cardiac disorders; these patients were from various parts of Greece. Subjects were classified as LMCAD group, n = 133; with angiographically documented left main coronary artery disease, MPCAD group (more peripheral coronary artery disease), n = 241 and Control group, n = 97. Control group was recruited from Onassis Cardiac surgery Center personnel and teachers from TEI schools (without major risk factors) who were self reported as healthy.

Major classical CAD risk factors were evaluated according to the National Cholesterol Education Program - Adult Treatment Panel III guidelines. diabetes mellitus was defined as fasting glucose > 126 mg/dl (7 mmol/L) or currently receiving antidiabetic medication; hypercholesterolaemia was defined as total cholesterol > 170 mg/dl (4.4 mmol/L) for subjects with CAD; hypertension was defined as BP> 140/90 mmHg or currently on treatment with antihypertensive medication. All patients who recruited in the study gave informed consent. The Onassis Cardiac Surgery Center ethics committee approved the protocol of this study.

### Angiographic Evaluation

Coronary angiograms were scored systematically and in random order by a single, experienced, interventional cardiologist. LMCAD was defined as a lesion compromising the lumen by >30% proximal to the bifurcation, including ostial stenosis. Lesions compromising the lumen by >50% further from LM were defined as MPCAD.

### CETP Genotyping

After the recruitment of the study population, genotyping of CETP polymorphisms (TaqIB and I405V) was performed by polymerase chain reaction (PCR) and restriction fragment length polymorphism analysis as described previously by others [20,21]. Briefly, each PCR reaction was performed using 500 ng of genomic DNA in a volume of 25 μl containing 50 mM KCl, 10 mM TRIS HCl (pH 8.8), 200 μM dNTPs, 1.0-1.5 mM MgCl_2_, 12.5-25 pmol of each primer and 0.75 U of Taq polymerase (Keymed S.r.I., Rome, Italy). The intron 1 region containing the TaqIB polymorphism was amplified using the forward oligo 5'-CAC TAG CCC AGA GAG GGA GTG CC-3' and the reverse oligo 5'-CTG AGC CCA GCC GCA CAC TAA C-3', giving a fragment of 535 bp length [21]. The exon 14 region containing the I405V polymorphism was amplified using the forward oligo 5'-TAT TTT TTT CAC GGA TGG GCA-3' and the reverse oligo 5'-TTG ACT GCA GGA AGC TCT GGC-3', giving a fragment of 142 bp length [20]. For the TaqIB polymorphism, the PCR conditions were an initial denaturation at 95°C for 5 min, followed by 30 cycles at 95°C for 30 sec, 65°C for 30 sec and 72°C for 30 sec and finally at 72°C for 7 min. For the I405V polymorphism, the PCR conditions were 95°C for 5 min, 60°C for 1 min and 72°C for 1 min for one cycle, and subsequently 35 cycles at 95°C for 30 sec, 60°C for 30 sec and 72°C for 30 sec and finally at 72°C for 5 min. For the detection of TaqIB polymorphism 10 μl of the PCR product were digested with 5 U of TaqI (New England Biolabs, Frankfurt, Germany) at 65°C overnight, giving 174bp and 361 bp fragments in presence of the TaqI site. For the detection of I405V polymorphism 8 μl of the PCR product were digested with 5 U of MspI (New England Biolabs, Frankfurt, Germany) at 37°C overnight, giving 121 bp and 21 bp fragments in presence of the less common V allele.

### Biochemical Analysis

Plasma total cholesterol, triglycerides and HDL-C were measured using enzymatic colorimetric methods on a Roche Integra Biochemical analyzer with commercially available kits (Roche Diagnostics Gmbh, Hannheim, Germany). The serum LDL-C levels were calculated using the Friedewald formula [22] only in patients with triglyceride levels <400 mg/dl (<4.5 mmol/l). Lipoprotein (a), apolipoprotein A and apolipoprotein B was measured by nephelometry (Nephelometer: BN-100, Behring, Germany). Blood glucose was measured by the hexokinase method with a Dade Behring reagent on a Dimension (Dade Behring) instrument. All samples were analyzed within 24 h.

### Statistical Analysis

The results are expressed as mean ± standard deviation (SD) or as median and interquartile range (IQR) according to normality of continuous variables. All qualitative variables are presented as absolute or relative frequencies. Differences in lipid levels for the various genotypes were evaluated with one - way analysis of variance (ANOVA) or its non-parametric analogue Kruskal - Wallis H statistic. The Student's t-test or its non-parametric equivalent Mann-Whitney U test was used to compare the continuous variables between the two groups studied. The Pearson's chi-square test was employed for the categorical variables. The allele frequencies for both TaqIB and I405V were found in Hardy-Weinberg equilibrium. All tests were two-tailed and statistical significance was established at 5% (p<0.05). Data were analysed using Stata ™ (Version 10.1 MP, Stata Corporation, College Station, TX 77845, USA).

## Results

Demographic and clinical characteristics of the study population and biochemical markers, including lipid profile for the two CETP polymorphisms are shown in Table 1. MPCAD and LMCAD patients do not differ according to demographic data (Table 1). Age seems to be an exception, since LMCAD patients were older than MPCAD patients (p = 0.004). A comparison of the lipid profile between the two groups revealed differences in HDL-C and Lipoprotein (a) levels, with the LMCAD group sustaining higher levels compared to the MPCAD group (Table 1). The Control group comprised 97 individuals (80% women) mean age 59 (18) years old. Demographic, clinical and biochemical variables are significantly different between LMCAD or MPCAD groups when compared to Controls [Body mass index: 24 (3.1) kg/m2, waist: 85 (9.3) cm and HDL-C: 67 (30) mg/dl]. The allele frequencies for both TaqIB and I405V are shown in Table 2.

**Table 1:** Characteristics of the MPCAD and LMCAD groups

Variable	MPCAD (n = 241)	LMCAD (n = 133)	P*
Demographic data			

Sex (M/F)	194/31 (86%/14%)	121/10 (92%/8%)	0.08
Age (ys)	62 (10)	65 (12)	0.004
Waist (cm)	104 [84-107]	104 [95-112]	0.55
BMI (Kg/m^2^)	27 (3.5)	28 (3.8)	0.53

Lipid profile (in mg/dl)			

Total Cholesterol	240 (61)	244 (60)	0.75
Triglycerides	140 [106-207]	135 [97-181]	0.17
HDL cholesterol	37 [31-44]	40 [35-47]	0.01
LDL cholesterol	151 (46)	150 (36)	0.86
Apolipoprotein A	111 [97.7-130.8]	119 [102-139.3]	0.24
Apolipoprotein B	96 [78-116]	93 [80-116]	0.83
Lipoprotein(a)	15 [10-31]	24 [18-73]	0.03

Clinical characteristics			

Smoking (yes/no)	66/142 (32%/68%)	55/87 (30%/70%)	0.8
Diabetes Mellitus	87/135 (39%/61%)	57/65 (47%/53%)	0.18
Hypertension (yes/no)	156/69 (69%/31%)	92/31 (75%/25%)	0.28

Biochemical markers			

Glucose (mg/dl)	105 [94-129]	103 [94-116]	0.18
Creatinine (mg/dl)	1 [0.9-1.2]	1 [0.9-1.2]	0.19
Ht (%)	41 (4.7)	41 (4.8)	0.86

MPCAD = more peripheral coronary artery disease, LMCAD = left main coronary artery disease

*Statistical tests performed: Student's t-test or its non-parametric equivalent Mann-Whitney U test was used for comparison of continuous data - Pearson's chi-square test was employed for comparison of categorical variables.

Data are expressed as mean ± standard deviation (SD) or as median and interquartile range (IQR) according to normality of continuous variables. All qualitative variables are presented as absolute and relative frequencies.

**Table 2:** Allele frequencies of studied population

Allele frequencies	Controls (n = 97)	MPCAD (n = 241)	LMCAD (n = 133)
I	0.69	0.68	0.63
V	0.31	0.32	0.37

B1	0.46	0.60	0.62
B2	0.54	0.40	0.38

MPCAD = more peripheral coronary artery disease, LMCAD = left main coronary artery disease

There is no difference in CETP allele frequency or genotype distribution among LMCAD and MPCAD patients (Table 3) although there is difference between LMCAD and Controls (p = 0.001). No such difference was detected when compared the I405V polymorphisms among the same groups (Table 3).

**Table 3:** Distribution of CETP I405V (II, IV, VV) and TaqIB (B1B1, B1B2, B2B2) genotypes on studied population

CETP genotypes	Controls (n = 97)	MPCAD (n = 241)	LMCAD (n = 133)
I405V			

II	40 (42%)	111 (46%)	51 (38%)
IV	52 (54%)	105 (44%)	66 (50%)
VV	4 (4%)	25 (10%)	16 (12%)
		(P = 0.08*)	(P = 0.12*)

TaqIB			

B1B1	22 (23%)	81 (34%)	45 (34%)
B1B2	45 (47%)	128 (53%)	74 (56%)
B2B2	29 (30%)	32 (13%)	14 (10%)
		(P = 0.001*)	(P = 0.001*)

MPCAD = more peripheral coronary artery disease, LMCAD = left main coronary artery disease

*Comparisons were made between LMCAD group vs Control group and MPCAD group vs Control group. Pearson's chi-square test (or Fisher's exact test when appropriate).

Specifically, patients with LMCAD and B1B1 genotype were more frequent present compared to Controls (33.8% vs 22.9%, respectively). The frequency of B2B2 genotype was 3 times lower in the LMCAD group compared to Controls (10.5% vs 30.2%, respectively). In the LMCAD group the frequency of B1 allele compared to Controls was higher (Table 2).

Blood lipid levels in LMCAD and MPCAD groups did not differ according to I405V polymorphism. Similarly, the relationship between TaqIB gene polymorphism and the LMCAD group was independent of lipid profile, with the exception of apolipoprotein A (p = 0.028). However, TaqIB gene polymorphism seemed to differ in the MPCAD group according to HDL-C, apolipoprotein A and lipoprotein(a) concentrations (p = 0.016, p = 0.003, p = 0.03, respectively).

## Discussion

To our knowledge, this is the first genetic CETP gene polymorphisms association involving CAD study stratifying on localization of CAD and particularly on LM.

The frequency of TaqIB polymorphisms in our population was similar to that reported for Greeks and other Caucasian populations (B1:55%, B2:45%, I:65%, V:35%) [23,24] suggesting that our study population is not genetically different from other cohorts.

It was already suggested by others [25] and us [26] that there may be a link between CETP polymorphisms and severity of CAD. Our previous study [26] reported that the I405V polymorphism of CETP gene was linked with severity of coronary artery stenosis estimated by the Gensini Score (defines narrowing or occlusion of the lumen of the coronary arteries from 1 to 32 score; the LM has the highest location score). Thus, we thought, that the association of CETP polymorphisms will be more pronounced in LM disease and will allow us to distinct from peripheral CAD. However, we did not found any difference in genotypic frequencies of TaqIB or I405V polymorphisms between LMCAD and MPCAD groups. Although, the genotypic difference concerning TaqIB polymorphism was found between Controls and LMCAD groups. This suggested that the frequency of TaqIB polymorphism may be associated with disease severity [MPCAD (moderate disease) and LMCAD (severe disease)]. Kuivenhoven et al [24] investigated the association between TaqIB polymorphism and the progression of coronary atherosclerosis in men. Their results indicated that B1 allele was associated with the increased progression of atherosclerosis in a dose-dependent manner [24]. Similarly to our results in which we found association of B1 allele only with LMCAD and not with MPCAD. Also, Dandona et al suggested a gene dosage of the common variant 9p21 locus and the severity of coronary atheromatous burden [27]. Furthermore, Fischer et al [16] reported that 9 of 12 monozygotic twin pairs displayed concordance for LM lesions, whereas only 3 of 12 were concordant for MP lesions [28,29], suggesting genetically involvement. Capodanno et al [30] investigated the epidemiology and the clinical impact of different anatomical phenotypes of the LM coronary artery and Iwasaki et al [31] investigated the distribution of coronary atherosclerosis in patients with CAD. They findings suggested that LM phenotypes are more likely to present with atherosclerotic disease and significant stenosis [30] and are particularly heritable [31]. The cause for different heritability estimates of LM and MP lesions remains unclear. Firstly, it could be related to the different ontogenetic determination of LM and MP sites. The coronary vessels develop from the blood islands [32-35]. The LM parts of the coronary arteries develop as buds on the walls of the truncus arteriosus and the MP portion develops as a subepicardial vascular network [16]. Secondly, it could be related to anatomy of the LM coronary artery, which is composed from three parts [ostium, a body and a distal portion or bifurcation [36]. The ostium has a greater proportion of smooth muscle and elastic tissue than the rest of the coronary vessels [37]. This suggest that although the LM disease seems to be influenced by genetic factors in a great extent the CETP gene polymorphisms, involved in lipid metabolism, may not be the one to differentiate between the LM and MPCAD.

This study has limitations. The sample of the study is small due to the frequency of disease (LM disease has been found in 3% to 5% of all patients who undergo coronary angiography [38]), therefore, this type of study is rarely conducted and cannot be performed in larger and broader epidemiological studies. The fact that this study was conducted in a Mediterranean country should not be neglected in terms of the high prevalence of smokers (nearly 50% in general population and 10-30% in study cohort), compared with some European countries [39]. Several ethnic differences which may play role in CAD risk have also been documented by our group [39]. No gender evaluation was performed due to small number of women in the cases groups.

## Conclusions

LM disease seems to be heritable to a considerable extent. This means that in healthy relatives of affecting families will have an increased risk for severe coronary events. Thus, any information to detect the asymptomatic relatives of these patients can be useful for primary prevention. In this study, the findings indicate that the TaqIB polymorphism may have potential importance in screening individuals at high risk for developing CAD. However, this polymorphism cannot distinguish between LMCAD and MPCAD. No any association was found between I405V polymorphism and CAD. However, further prospective investigations in larger populations are required to confirm these findings.

## Competing interests

The authors declare that they have no competing interests.

## Authors' contributions

GK conceived of the study, and participated in its design and coordination. SM, EP and SP collected the blood samples and evaluated the patient's medical history. IV evaluated coronary angiographs. VK and AM carried out the molecular genetic studies and drafted the manuscript. AK participated in the design of the study and performed the statistical analysis. DD participated in revising the manuscript critically for important intellectual content. HD and VG participated in the study design and its coordination. All authors read and approved the final manuscript.
